# Severe Metformin-Associated Lactic Acidosis Managed Without Dialysis in an Elderly Patient With Multiple Comorbidities: A Case Report

**DOI:** 10.7759/cureus.89686

**Published:** 2025-08-09

**Authors:** Hironobu Sasaki, Kazuma Yagi, Yohei Uratsuji, Tatsuya Fujii, Masaki Koide

**Affiliations:** 1 Department of Internal Medicine, Sainokuni Higashiomiya Medical Center, Saitama, JPN; 2 Department of Emergency and Critical Care Medicine, Sainokuni Higashiomiya Medical Center, Saitama, JPN

**Keywords:** acute kidney injury, advance care planning, critical care, diabetes, lactic acidosis, metformin-associated lactic acidosis, metformin toxicity, non-dialytic management

## Abstract

Metformin-associated lactic acidosis (MALA) is a rare but potentially life-threatening adverse effect. Extracorporeal treatment is generally recommended for severe MALA, defined by a lactate concentration >20 mmol/l, arterial pH ≤7.0, shock, or decreased level of consciousness. We report a case of an 84-year-old Japanese man with autoimmune pancreatitis and type 2 diabetes who developed severe MALA while continuing metformin despite prolonged poor oral intake, and was successfully treated without dialysis. On admission, the patient presented with hypothermia, shock, acute kidney injury (serum creatinine 5.93 mg/dl), hyperkalemia (serum potassium 7.7 mEq/l), and lactic acidosis (arterial pH 6.962, lactate 17 mmol/l), which later worsened to a lactate level of 26 mmol/l. While meeting the criteria for emergency dialysis, renal replacement therapy was withheld in accordance with his advance care planning. The patient received intensive supportive therapy, including aggressive intravenous fluid administration, vasopressor support with noradrenaline and vasopressin, hydrocortisone, vitamin B1, and sodium bicarbonate supplementation. Acidosis resolved by 36 hours after admission, and the lactate level decreased to below 5 mmol/l by 44 hours. He was discharged without sequelae on day 31. This case suggests that even in elderly patients with severe MALA, non-dialytic treatment may be effective when intensive and targeted supportive care is provided.

## Introduction

Metformin is an insulin-sensitizing agent that primarily acts by suppressing hepatic gluconeogenesis and enhancing insulin sensitivity in peripheral tissues [1,2]. Owing to its low cost and robust evidence supporting the reduction in macrovascular complications in patients with type 2 diabetes, metformin has long been recommended as a first-line therapy in major clinical guidelines [3,4]. In Japan, the maximum approved daily dose of metformin was increased from 750 mg to 2250 mg in 2010, and its prescription rate has steadily increased over the past two decades [5]. However, metformin has been associated with lactic acidosis, a rare but potentially fatal adverse effect. Metformin-associated lactic acidosis (MALA) is generally defined by a blood lactate level >5 mmol/l and an arterial pH <7.35 in the context of current or recent metformin use [6]. The estimated incidence of MALA is approximately six cases per 100000 patient-years [7]. Although earlier studies reported mortality rates as high as 50%, recent data still show a considerable mortality rate of up to 25% [8]. Since metformin is excreted unchanged by the kidneys, impaired renal function may lead to its accumulation, theoretically increasing the risk of MALA. Therefore, metformin is contraindicated in patients with an estimated glomerular filtration rate of <30 ml/min/1.73 m^2^ [9]. Hemodialysis is effective in removing metformin, correcting metabolic acidosis and hyperlactatemia, and supporting renal function, thereby contributing to favorable outcomes [7]. Extracorporeal treatment (e.g., hemodialysis or continuous renal replacement therapy) is recommended for severe MALA, defined by a lactate concentration >20 mmol/l, arterial pH ≤7.0, shock, or decreased level of consciousness [10]. Here, we present a rare case of an elderly patient who developed severe MALA complicated by shock, pneumonia, and acute kidney injury. Despite fulfilling the criteria for extracorporeal treatment, the patient was successfully managed without hemodialysis through intensive supportive care. Importantly, we were able to closely monitor the course of the acid-base parameters and lactate levels through frequent arterial blood gas analyses.

## Case presentation

An 84-year-old Japanese man weighing 37.6 kg (BMI 14.9 kg/m^2^), with a 14-year history of autoimmune pancreatitis treated with prednisolone (7.5 mg/day) for at least 10 months (prior dose unknown), was taking metformin (1000 mg/day) and linagliptin (5 mg/day) for type 2 diabetes. The patient had experienced reduced oral intake for one month before admission, but had continued taking his medications. On the morning of admission, the patient experienced a decreased level of consciousness while independently propelling a wheelchair to have breakfast. Upon contact with the emergency crew, the blood glucose level was found to be below 20 mg/dl, and glucose was administered before the patient was transported to our hospital. At the time of presentation, the patient was found to be hypothermic, with a body temperature of 32.8°C, and in a state of shock, with a blood pressure of 85/56 mmHg. Chest computed tomography revealed bilateral lower lobe pneumonia. Although his baseline serum creatinine (Cr) level was approximately 1 mg/dl, blood tests on arrival revealed acute kidney injury with a serum Cr level of 5.93 mg/dl. Hyperkalemia was also present, with a serum potassium level of 7.7 mEq/l. Therefore, 0.85 g of calcium gluconate was administered intravenously, and glucose-insulin therapy was conducted with 20 g of glucose and 2 units of regular insulin in the emergency room. Additionally, arterial blood gas analysis revealed a pH of 6.962 and a lactate level of 17 mmol/l, indicating lactic acidosis. The anion gap was elevated at 30.9 mmol/l, consistent with high anion gap metabolic acidosis. Considering ongoing metformin use, the patient was diagnosed with MALA and admitted to the intensive care unit (ICU), and metformin was subsequently discontinued. The admission laboratory findings are shown in Table 1.

**Table 1:** Laboratory data on admission Alb, Albumin; ALP, Alkaline phosphatase; ALT, Alanine aminotransferase; APTT, Activated partial thromboplastin time; AST, Aspartate transaminase; BE, Base excess; BUN, Blood urea nitrogen; Ca, Calcium; CK, Creatine kinase; Cl, Chloride; Cr, Creatinine; CRP, C-reactive protein; D-Bil, Direct bilirubin; FDP, Fibrin degradation product; ɤ-GTP, Gamma-glutamyl transferase; Hb, Hemoglobin; HbA1c, Glycated hemoglobin A1c; HCO_3_^-^, Bicarbonate; Ht, Hematocrit; K, Potassium; LDH, Lactate dehydrogenase; MCH, Mean corpuscular hemoglobin; MCHC, Mean corpuscular hemoglobin concentration; MCV, Mean corpuscular volume; Mg, Magnesium; Na, Sodium; P, Phosphorus; P-AMY, Pancreatic amylase; PaCO_2_, Partial pressure of arterial carbon dioxide; PaO_2_, Partial pressure of arterial oxygen; Plt, Platelet; PT-INR, Prothrombin time-international normalized ratio; RBC, Red blood cell; T-Bil, Total bilirubin; TP, Total protein; WBC, White blood cell.

	Result	Reference range
Blood biochemistry		
TP (g/dl)	5.1	6.6-8.1
Alb (g/dl)	2.4	4.1-5.1
T-Bil (mg/dl)	0.1	0.4-1.5
D-Bil (mg/dl)	0.1	0-0.4
AST (U/l)	20	13-30
ALT (U/l)	12	10-42
LDH (U/l)	149	124-222
ALP (U/l)	46	38-113
ɤ-GTP (U/l)	16	13-64
CK (U/l)	56	59-248
P-AMY (U/l)	95	0-65
BUN (mg/dl)	112.2	8-20
Cr (mg/dl)	5.93	0.65-1.07
Na (mEq/l)	133	138-145
K (mEq/l)	7.7	3.6-4.8
Cl (mEq/l)	97	101-108
Ca (mg/dl)	10.8	8.8-10.1
P (mg/dl)	6.6	2.7-4.6
Mg (mg/dl)	3.7	1.8-2.6
CRP (mg/dl)	3.89	0-0.14
Glucose (mg/dl)	178	73-109
HbA1c (%)	7.2	4.9-6.0
Peripheral blood		
WBC (/mm^3^)	8500	3300-8600
RBC (× 10^4^/mm^3^)	329	435-555
Hb (g/dl)	10.4	13.7-16.8
Ht (%)	34.1	40.7-50.1
MCV (fl)	103.6	83.6-98.2
MCH (pg)	31.6	27.5-33.2
MCHC (%)	30.5	31.7-35.3
Plt (× 10^4^/mm^3^)	23.8	15.8-34.8
Blood coagulation test		
PT-INR	1.44	0.85-1.15
APTT (second)	35.2	24.0-34.0
FDP (µg/ml)	3.5	0-5.0
Arterial blood gas analysis		
pH	6.962	7.38-7.46
PaCO_2_ (mmHg)	19.9	32-46
PaO_2_ (mmHg)	78.6	74-108
HCO_3_^-^ (mmol/l)	4.5	21-29
BE (mmol/l)	-25.8	-2-2
Anion gap (mmol/l)	30.9	
Lactate (mmol/l)	17	0.5-1.6

Given the diagnosis of severe MALA, the need for emergency dialysis was considered upon admission. However, renal replacement therapy was withheld in accordance with the patient’s previously expressed wishes to forgo invasive treatments, including dialysis. This decision was made in consultation with his family and aligned with his advance care planning (ACP), a process in which patients express their preferences for future medical treatment in anticipation of a time when they may no longer be able to communicate them. Supportive care was therefore prioritized.

**Figure 1:**
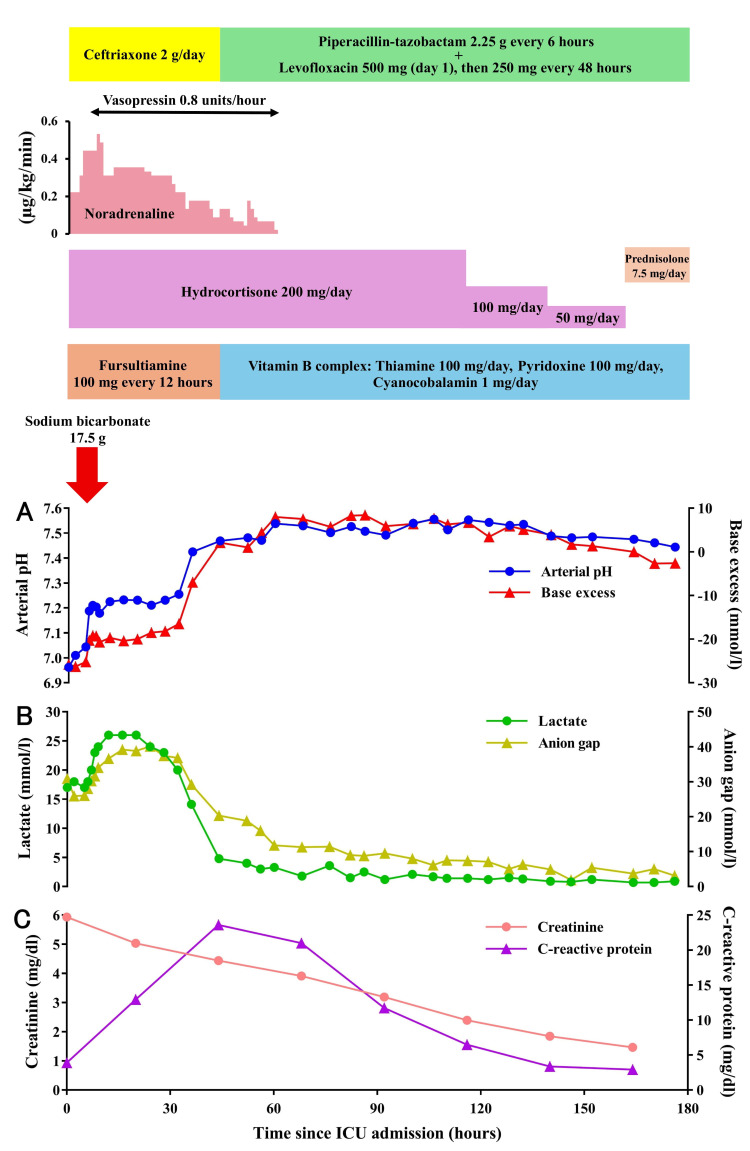
Clinical course of the patient with metformin-associated lactic acidosis since admission to the intensive care unit (ICU) A: Temporal changes in acid-base parameters (arterial pH and base excess); B: Time course of lactate levels and anion gap; C: Temporal changes in renal function (serum creatinine) and inflammatory response (C-reactive protein).

On the day of admission, 4000 ml of intravenous fluids, primarily Ringer’s solution, were administered to improve renal function and blood pressure. Noradrenaline and vasopressin infusions were initiated to maintain hemodynamic stability. Considering the history of prednisolone use at 7.5 mg/day for over 10 months, relative adrenal insufficiency was suspected. Although adrenal function was not formally evaluated, hydrocortisone was empirically administered at a dose of 200 mg/day. In consideration of the possibility that long-standing appetite loss had led to thiamine deficiency, which may have contributed to the development of lactic acidosis in addition to the effects of metformin, intravenous administration of vitamin B1 (fursultiamine 100 mg every 12 hours) was also initiated. To correct acidosis, 17.5 g of sodium bicarbonate was administered via intravenous infusion, resulting in an increase in pH and base excess (Figure 1A).

In contrast, both the lactate level and the anion gap continued to rise after admission, with lactate peaking at 26 mmol/l between 12 and 20 hours post-admission (Figure 1B). However, an additional 2500 ml of intravenous fluids was administered on day two of hospitalization to improve the circulatory status further, which led to a decrease in the lactate levels and the anion gap. Arterial blood pH rose above 7.35 by 36 hours post-admission, and the lactate level had dropped to 4.8 mmol/l by 44 hours, indicating successful resolution of lactic acidosis. Treatment for pneumonia was initially started with ceftriaxone, but due to identification of Enterobacter cloacae, antibiotics were switched to piperacillin-tazobactam plus levofloxacin, which led to a decrease in CRP levels (Figure 1C). With circulatory status improving, both noradrenaline and vasopressin were discontinued by day three of hospitalization, and serum Cr levels showed a gradual improvement over time (Figure 1C). As the patient's overall condition stabilized, hydrocortisone was gradually tapered. The patient was transferred out of the ICU on day eight of hospitalization and subsequently continued rehabilitation, leading to discharge on day 31 of hospitalization.

## Discussion

MALA is a rare condition, but once it occurs, it can be life-threatening. Metformin inhibits mitochondrial respiratory-chain complex I, which leads to reduced hepatic gluconeogenesis. As a result, substrates such as lactate are less efficiently utilized for glucose production, potentially causing lactate accumulation [11]. MALA typically warrants caution in the presence of conditions that reduce metformin clearance, such as renal impairment, or impair lactate clearance, such as hepatic dysfunction. It is also a concern in situations that increase lactate production, including sepsis, heart failure, impaired tissue perfusion, or hypoxia. Furthermore, severe dehydration, shock, alcohol consumption, and advanced age can be contributing risk factors [11]. In this case, multiple factors may have contributed to the onset of MALA, including acute renal failure, infection-related shock, long-term use of corticosteroids, and malnutrition.

In the treatment of MALA, extracorporeal treatment is recommended in severe cases with marked hyperlactatemia and progressive metabolic acidosis [10]. Observational studies have shown that initiating renal replacement therapy within six hours of onset and selecting hemodialysis significantly reduces 30-day mortality [12]. Dialysis performed until normalization of serum lactate, bicarbonate, and potassium levels has been associated with lower mortality than previously reported [13]. In the present case, despite the severity of the condition warranting dialysis, the patient was successfully managed without undergoing dialysis. To the best of our knowledge, case reports of MALA successfully managed without the need for dialysis are rare. Table 2 summarizes a comparison between the present case and previously reported cases [14,15].

**Table 2:** Comparison of previously reported cases of metformin-associated lactic acidosis managed without dialysis ICU, Intensive care unit; PT-INR, Prothrombin time-international normalized ratio.

	Present case	Case 1 (İnal et al. [14])	Case 2 (Fukuda et al. [15])
Age	84	65	77
Sex	Male	Male	Male
Metformin dose (mg/day)	1000	2000	250
Pre-treatment arterial pH	6.962	7.05	7.295
Pre-treatment lactate level (mmol/l)	17	19.4 (175 mg/dl)	5.76
Serum creatinine at admission (mg/dl)	5.93	2.9	2.52
Blood pressure at admission (mmHg)	85/56	100/70	146/58
Dialysis	Not performed due to advance care planning	Planned but withheld due to high PT-INR	Considered, but not performed due to rapid clinical improvement
Post-admission treatment	intravenous fluids, vasopressors, corticosteroids, vitamin B1, sodium bicarbonate, and antibiotics	intravenous fluids, fresh frozen plasma, vitamin K, sodium bicarbonate, and antibiotics	intravenous fluids
Clinical Course	ICU discharge on day 8; hospital discharge on day 31	hospital discharge on day 8	ICU discharge within 12 hours; hospital discharge on day 5

The present case involved a patient older than those in prior reports, with a markedly lower arterial pH and significantly elevated serum Cr, indicating more severe acidosis and renal impairment. Unlike the earlier cases, this patient also presented with hypotension at admission, suggestive of shock. The longer ICU stay and hospitalization, compared to previous reports, likely reflect the greater severity of illness.

Several factors may have contributed to the favorable outcome in this case, despite the severity of MALA and the absence of dialysis. First, the prompt and appropriate initiation of supportive therapy likely played a crucial role. In particular, the improvement of circulatory dynamics through the administration of appropriate volumes of intravenous fluids may have contributed to the recovery of acid-base balance [16], as well as to the reduction of the anion gap. Additionally, the maintenance of blood pressure with vasopressors likely facilitated the clearance of lactate. In the combined use of noradrenaline and vasopressin, lactate clearance has been shown to improve significantly compared to noradrenaline alone [17]. In the present case as well, although lactate levels were increasing prior to the addition of vasopressin, they began to decrease after its initiation. Second, considering the possibility of relative adrenal insufficiency due to long-term corticosteroid use, hydrocortisone was administered, which may have played an important role in stabilizing circulatory dynamics. Indeed, appropriate hydrocortisone hastens shock resolution by reversing adrenal insufficiency [18]. Furthermore, thiamine (vitamin B1) supplementation was administered because thiamine deficiency, which may have been caused by prolonged loss of appetite, is known to exacerbate lactic acidosis [19]. While vitamin B1 levels were not measured in this case, this intervention may have contributed to the improvement in lactate accumulation. Despite meeting the criteria for dialysis due to its severity, recovery was achieved without it through intensive medical management and frequent monitoring with arterial blood gas analysis. This case suggests that even when dialysis cannot be performed due to ACP, adequate supportive therapy and careful monitoring may still enable a favorable outcome.

## Conclusions

Our case suggests that, in the context of severe MALA, even in elderly patients with multiple comorbidities, including acute kidney injury and pneumonia, non-dialytic management may be considered as a potential option in exceptional circumstances. However, dialysis remains the standard of care, given its role in rapidly correcting acidosis and removing metformin. Individualized treatment approaches that consider the patient’s clinical background and preferences, such as ACP, are essential. Although standard criteria may indicate dialysis, favorable outcomes might still be achieved with non-dialytic therapies in select cases.

To prevent this rare but serious complication, appropriate prescribing of metformin based on individual risk profiles is essential. Indeed, given the risks associated with metformin use during acute illnesses, especially in elderly patients or those with renal impairment or infection, clinicians should exercise increased vigilance and consider temporary discontinuation of metformin in such situations. Nevertheless, since severe cases may still occur, further accumulation of such cases is warranted to better define the indications for dialysis.
